# Impact of postdural puncture headache after diagnostic lumbar puncture 

**Published:** 2014

**Authors:** Ali Jabbari, Mohammad Reza Hasanjani Roushan

**Affiliations:** 1 Department of Anesthesiology and Intensive Care, Golestan University of Medical Sciences, Gorgan, Iran.; 2 Infectious Diseases and Tropical Medicine Research Center, Babol University of Medical Sciences, Babol, Iran.

**Sir**

Lumbar puncture (LP) is an essential medical procedure for several clinical conditions. It is an invasive procedure by which physicians can provide sample of cerebrospinal fluid through a needle inserted into the lower lumbar area for diagnostic purposes (meningitis or subarachnoid hemorrhage). Injection of medications into the cerebrospinal fluid "intrathecally", particularly for spinal anesthesia which is another important use of LP. Sometimes this procedure is indicated for pain management and chemotherapy or rarely for treatment of "therapeutic lumbar puncture" to relieve increased intracranial pressure (1, 2). The most common purpose for a lumbar puncture is anesthesia setting for neuroaxial block and collecting cerebrospinal fluid in cases suspected for meningitis. This is the most reliable method for confirming meningitis or exclusion of a possible life-threatening but highly treatable condition. Examination of the CSF can be also helpful for other clinical situations such as detecting the presence of malignant cells (1, 3). 

Despite a relatively safe procedure, but performing LP may be associated with several adverse events including postdural (post-lumbar or post-spinal) puncture headache (PDPH) (2, 3). Physicians who frequently perform LP for diagnostic or therapeutic purpose may encounter patients at risk of developing PDPH. Although the possibility of developing PDPH is low but physicians have concerns because they are not able to accurately diagnose PDPH and get familiar with the available therapies. Occasionally, there are techniques that physicians should utilize in the performance of lumbar punctures to minimize the incidence of PDPH (1, 2, 4). Nevertheless, this is inordinately concerning the patients (1).

**Definition and diagnosis: **PDPH is defined as any headache after a lumbar puncture that worsens within 15 minutes of sitting or standing and is relieved within 15 minutes of lying down (5). The hallmark of PDPH is a constant headache that exacerbates in the upright position and improves when lying down and resolves spontaneously within five to seven days. Ninety percent of PDPHs occurs within three days of the procedure and 66% starts in the first 48 hours (4). The International Headache Society has defined PLPH as a “headache that develops within 5 days of dural puncture and resolves within 1 week spontaneously or within 48 hours after effective treatment of the spinal fluid leak” (5). The headache is usually but not always bilateral and may be characterized by frontal, occipital, or generalized pressure or throbbing occurring when the patient is upright, and diminishing or resolving when supine. The headache worsens with head movement, coughing, straining, sneezing, and jugular venous compression. In one series, additional symptoms were present as, neck stiffness, nausea, vomiting, cochlear symptoms and ocular symptoms (1, 2, 4). Sometimes these signs and symptoms look like meningoencephalitis features. 

Commonly, physicians are not informed since PDPH can be easily diagnosed based upon the characteristic symptoms. Various imaging techniques have been described to help guide the diagnosis but are rarely needed except for excluding other causes of headache or for confirming serious fatal complications including subdural hematoma (1).

In clinical situations like undetected mass lesion or ventricular obstruction which performing LP pose patients at higher risk of developing cerebral herniation (2), the clinicians can use the clinical examination to guide the decision to obtain brain CT scan instead of universal neuroimaging. In the absence of any focal neurological findings, altered mental state or papilledema, the LP can be performed without first completing a CT scan (2, 4, 6).

**Salience about PDPH prevention: **PDPH occurs in up to 40% of patients when using the traditional bevel tip or Quincke needle. The risk of headache can be dramatically reduced by the use of atraumatic pencil point tip needles such as the Sprotte or Whitacre. However, many physicians may not be familiar with these needles and only 2% use them (7). By contrast, anesthesiologists commonly use the atraumatic needles. The recent published guidelines from the American Academy of Neurology have supported the use of atraumatic needles (8). 

Patient demographic risk factors include the following: female gender (twice as often as men); age (greatest in those 18-30 years of age and much less in children younger than 13 years and adults older than 65); lower body mass index; previous chronic or recurrent headache; and prior PDPH (1, 3, 4). The use of Quincke needle with a larger-diameter needle and conventional bevel tip could be likelihood of PDPH. However, smaller-diameter needles are not practical for diagnostic lumbar puncture because of the slow flow of CSF. Using the Sprotte needle, especially in patients at high risk for headaches (young) would make a dramatic reduction in headaches (1, 9). 

The risk of PDPH is reduced when the bevel is parallel rather than perpendicular. Fewer fibers are cut with parallel entry because the dural fibers run parallel to the long axis of the spine. Parallel position means that a plane passing through the flat part of the bevel, going through both edges of the bevel, is parallel to the long or vertical axis of the spine. The face of the bevel and the notch in the hub should point in the direction of the patient's side, not toward the patient's head or feet. Reinsertion of the stylet before needle removal should occur and patients do not require bed rest after the procedure (1, 9, 10). It is postulated that a strand of arachnoid could enter the needle along with the outflowing CSF and if the stylet is not replaced, the strand may be threaded back through the dura during removal of the needle, producing prolonged leakage of the CSF. By replacing the stylet before removing the needle, the strand would be pushed out and cut, reducing the risk of continued leakage and the resulting headache (9, 10). 

The conventional medical wisdom over the last several decades to avoid PDPH has used smaller gauge and modern needles which traumatize the dura less and make a smaller dura puncture (3, 9). We have observed higher prevalence of PDPH in our patients by using Quincke needle and recommend a 25G pencil point (Sprotte’s needle) needle which can be used successfully for diagnostic/ therapeutic LP, with significantly lower rate of PDPH (1, 9), and a small tendency towards a shorter duration of PDPH symptoms besides; there is the evidence that the “atraumatic” needle reduces the incidence of traumatic tap of vascular structures. There is a clear trend for a reduction of CSF red blood content in patients punctured with Sprotte’s needle (9, 10). Some investigators consider preventing programs by prescribing medications such as gabapentine, pergabaline, caffeine and corticosteroids (1), we do not suggest prescribing these to diagnostic LP.

**Treatment: **Most cases of PDPH are not severe, and so they respond quickly to conservative treatment. However, severe and debilitating cases may occur due to CSF leakage through the dural puncture site which may require more seriously (2, 3). Drug therapy is usually limited analgesics, caffeine, and bed rest. Following a lumbar puncture, persistent stay in bed in supine position for a period of time is usually recommended. Although, PDPH procedure bed rest is a wise advice but has shown no further necessity for prolong bed rest (1, 3). Unresponsive cases of post-lumbar puncture headaches may be a healing sign of more serious conditions like subdural hematoma, space occupation lesions and seizures or a presentation of a comorbid underlying disease which could be fatal (2, 3).

However, persistent and severe PDPH in patients without bacteremia, meningitis or any neurologic complications may require epidural blood patch. For this purpose, small amount of the patient's blood is injected into the epidural space near the site of the original puncture. The injected blood regulates and produces a clot which patches leak site (1, 4).
